# The collaborative mind: intention reading and trust in human-robot interaction

**DOI:** 10.1016/j.isci.2021.102130

**Published:** 2021-02-01

**Authors:** Samuele Vinanzi, Angelo Cangelosi, Christian Goerick

**Affiliations:** 1 Cognitive Robotics Lab, The University of Manchester, Manchester M13 9PL, UK; 2 AIST-AIRC, Tokyo, Japan; 3 Honda Research Institute Europe GmbH, Offenbach am Main 63073, Germany

**Keywords:** Artificial Intelligence, Human-Centered Computing, Human-Computer Interaction

## Abstract

Robots are likely to become important social actors in our future and so require more human-like ways of assisting us. We state that collaboration between humans and robots is fostered by two cognitive skills: intention reading and trust. An agent possessing these abilities would be able to infer the non-verbal intentions of others and to evaluate how likely they are to achieve their goals, jointly understanding what kind and which degree of collaboration they require. For this reason, we propose a developmental artificial cognitive architecture that integrates unsupervised machine learning and probabilistic models to imbue a humanoid robot with intention reading and trusting capabilities. Our experimental results show that the synergistic implementation of these cognitive skills enable the robot to cooperate in a meaningful way, with the intention reading model allowing a correct goal prediction and with the trust component enhancing the likelihood of a positive outcome for the task.

## Highlights

• Collaborative intelligence can be built through intention and trust estimation skills
• Intention reading enables the collaboration, giving clues on the pursued goals
• Trust estimation allows an agent to perform informed decision-making
• Trust-aware collaborations perform better when dealing with unskilled partners

## Graphical Abstract

**Figure:**
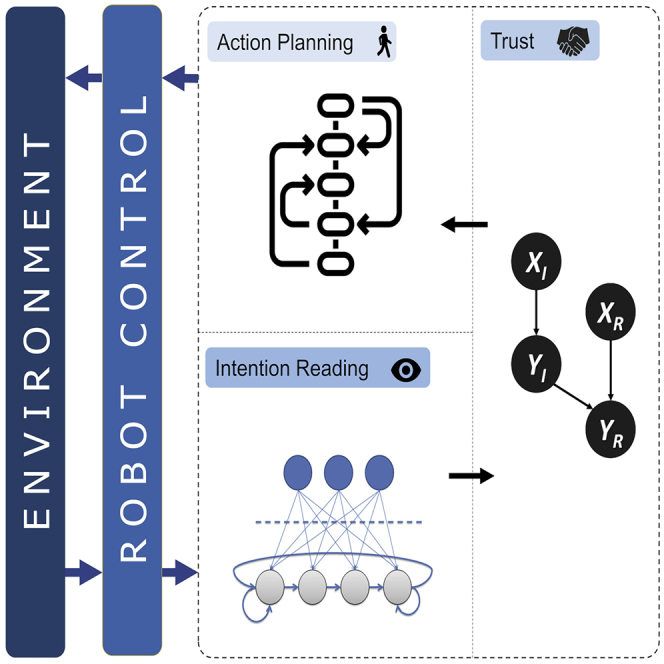

## Introduction

Human beings are social creatures held together by communal bonds and organized into complex social structures. This tendency to aggregation and to work as part of groups is not to be dismissed as a quirk but rather constitutes an important characteristic that has been proved being at least partially hardwired in our genes (Ebstein et al., 2010). The ability to collaborate with others to achieve common goals has been one of the key factors for our success as a species.

Researchers in the social sciences agree to distinguish collaboration from cooperation, as they represent two different types of interaction (Roschelle and Teasley, 1995). In particular, we refer to “cooperation” when the involved parties work toward a shared goal by solving sub-tasks individually and then assembling their partial results. In contrast, “collaboration” refers to the act of dividing the task among the participants, who then engage in a mutual, coordinated effort to solve the problem together. Given these definitions, the main difference between cooperation and collaboration is that the latter implies a deeper level of interaction, shared understanding, and coordination (Dillenbourg, 1999).

A body of scientific evidence points toward the early development of collaborative behaviors in human infants: the latter are, in fact, able to engage in coordinate actions as early as their first birthday. This ability continues to evolve through time and by experience, in parallel to their cognitive development, and by the 30th month of age, they become able to perform complementary actions (Henderson and Woodward, 2011).

Our hypothesis on collaborative intelligence stems from two statements. Bauer et al. (2008) break the collaboration process in a series of sequential tasks, namely perception, intention estimation, planning, and joint action. In other words, before an agent can collaborate with another, there is the need of recognizing the pursued goal and to select appropriate actions to maximize the chances of a successful outcome. Groom and Nass (2007) declare that trust is an essential component to successfully perform joint activities with common tasks. From these premises, we state that the two cognitive skills essential for successful collaboration are “intention reading” and “trust”.

We refer to intention reading as the ability to understand the goals of other agents based on the observation of their physical cues, for example, body posture, movements, and gaze direction. Generally speaking, humans do not perceive biological motion as meaningless trajectories through space but instead are able to view it in relation to an end objective (Malle et al., 2001). The cognitive process of estimating the intention is performed by dividing the observed continuous stream of actions in discrete intervals which are then individually decoded (Baldwin and Baird, 2001). By giving us the ability to understand what is happening around us, this ability lays the foundation of social awareness (Woodward et al., 2009), allowing us to reason about the behavior of other agents in our environment and acting accordingly.

Trust shares with intention reading the same importance in scaffolding our social abilities, as it affects every interaction we experience. Mayer et al. (1995) define it as the willingness of the trustor to rely on the actions of the trustee despite the former not having any control of the latter. The ability to correctly direct our trust has deep consequences on the success of our relationships, in our personal safety (Das and Teng, 2004) and in team cooperation (Jones and George, 1998).

Both these cognitive skills are not innate in humans, meaning that newborns do not automatically possess them. Instead, human phylogeny has provided each individual the tools to develop them in the scope of one's personal ontogeny, meaning that these traits will gradually arise during childhood and will refine themselves through social interactions and experiences, until reaching their full maturity. In particular, intention reading is facilitated in human beings by the mirror neuron system present in their brain (Rizzolatti and Craighero, 2004): a collection of neurons which activate both when the individual executes an action or when it observes a similar action being performed by someone else. By mapping the visual perception with the organism's own motor representation, this neurological system enables action understanding and imitation learning (Gallese and Goldman, 1998). This system is tuned by epigenetic processes during postnatal development (Ferrari et al., 2013), so it is correct to say that intention reading is perfected through experience; this is also confirmed by the fact that children are initially able to recognize biological motion, with time they start associating social cues such as biological motion and eye gaze (Tomasello et al., 2005) to goals and finally manage to understand the choice of plans (Woodward et al., 2009). In contrast, the developmental evolution of trust is still under debate. Erikson (1993) has theorized the stages of psychological development, the first of which is known as the “trust vs mistrust” stage that occurs around the second year of age: during this phase, the child's propensity to trust is directly influenced by the quality of cares he or she receives. This happens because infants depend entirely on their caregivers for sustenance, so if their needs are regularly satisfied, they will learn that the world is a secure and trustable place, or vice versa.

Both of these cognitive traits depend on a third one: theory of mind (ToM), the ability to understand that other beings around us possess different sets of mental states, such as beliefs, goals, and desires (Vanderbilt et al., 2011). Mastery of this capacity is a fundamental requirement for both the collaborative skills we are analyzing. In particular, intention reading can be performed only if it is possible to determine which desires are driving the actions of another agent, and trust can be estimated only if it is possible to compare beliefs and motivations to verify their alignment with one's owns (Premack and Woodruff, 1978). This dependency is emphasized by the fact that both these skills fully mature around the fifth year of age, which is also the same age at which ToM fully develops (Tomasello et al., 2005; Vanderbilt et al., 2011; Wellman et al., 2001).

Given the importance of collaborative behavior for humans, it seems natural to transpose its value to artificial agents, in particular to social robots which are expected to act in human-shaped environments interacting with us on a daily base. In particular, if we aim at designing robots able to blend themselves in our present and future societies, a strict requirement for them will be to adapt to our social expectations and fit in our natural environments. In other words, in a future where interactions between humans and robots will be more common, we do not want to robotize people, but we hope to make the minds of these mechanical companions a little more human. For this purpose, collaborative intelligence may be one of the most important skills for these agents to possess.

**Figure 1:**
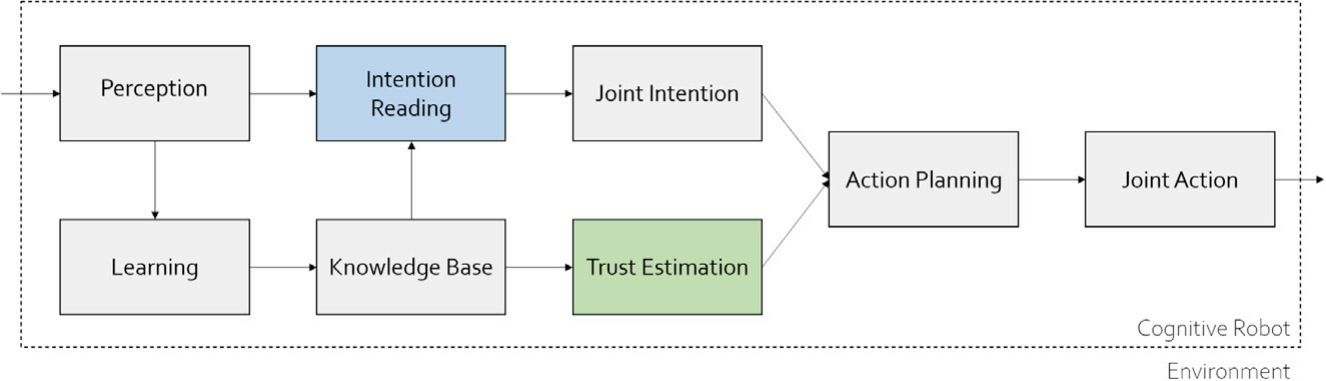
Overview of the mechanisms leading to joint action Expanded from Bauer et al. (2008) through the addition of trust estimation.

Collaborative intelligence, under a technical perspective, can be defined as a multi-agent system where each agent has the autonomy to contribute to a problem-solving network (Gill, 2012). For the purpose of this paper, we are interested in considering the special case of two agents, one human and a robot, which are collaborating to complete some task. In this work, we intend to expand the general collaboration architecture for cognitive robotics provided by Bauer et al. (2008) adding trust estimation between the intention reading and the action planning steps. Our proposed architecture is shown in Figure 1.

**Figure 2:**
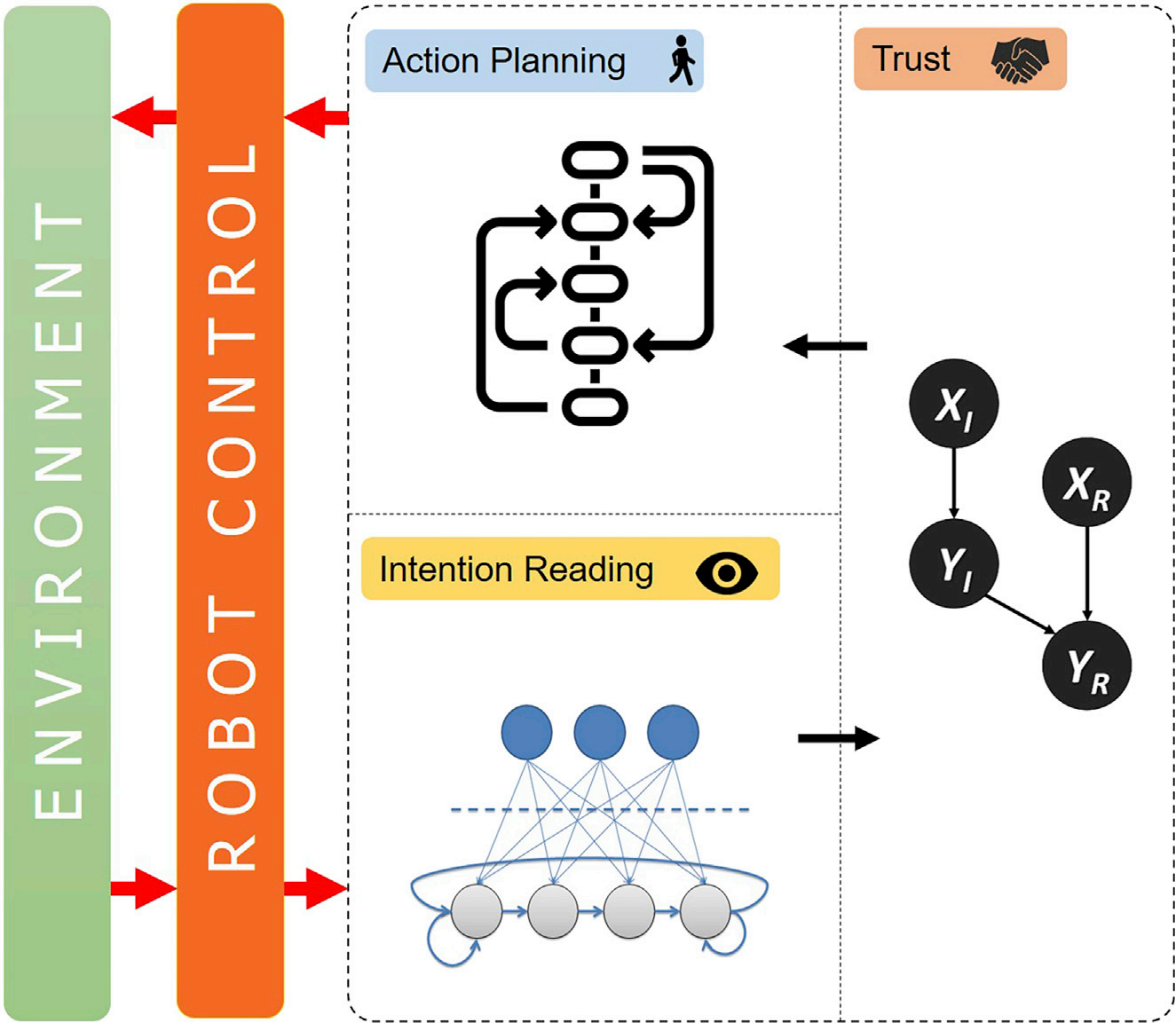
The proposed artificial cognitive architecture which integrates intention reading and trust mechanisms for the purpose of collaborative intelligence Please refer to the Transparent methods section of the Supplemental information for the detail of each component.

The scientific community has been investigating computational models for artificial intention reading for many years, as this is an important skill for collaborative machines (Vinanzi et al., 2019a). Some techniques seem to be more common than others, in particular hidden Markov models (Kelley et al., 2008) and Bayesian networks (BNs) (Dindo et al., 2015) seem to have gained a large consensus, as well as a wide range of machine learning methods such as neural networks (Singh et al., 2016) and support vector machines (Manzi et al., 2017). Hybrid approaches have also been investigated, for example, Granada et al. (1995) used a neural architecture to extract low-level features from camera images which are then used in a probabilistic plan recognizer. The use of embodied agents such as robots for the exploration of intention reading capabilities is promoted by Sciutti et al. (2015), who underline the importance of sharing the same action space with the human partner. Robots have in fact been successfully used to investigate intention understanding and sharing in turn-based games that possess a strong learning-by-demonstration aspect (Dominey and Warneken, 2011; Jansen and Belpaeme, 2006).

Trust has also been extensively researched in the context of human-robot interaction (HRI), the main reason being that the quality of the interaction is usually shaped by how trustworthy the robot appears to the human. This means that even a perfect machine will not be able to perform at its fullest if the human partner is not willing to trust its decisions and actions. This problem has generated a branch of research focused on determining which behavioral and esthetic elements of a robot can influence its perception from the people who interact with it, in other words there is a vast literature of human-centered trust in HRI (Floyd et al., 2014; Zanatto, 2019). Here, we propose that the opposite, i.e., the trustworthiness of a human estimated by a robot, is also fundamental during a collaborative activity: whereas a robot can fail, so can a person, and it is important to keep this in mind when performing decisions that will try to optimize the achievement of the shared goal. Unfortunately, literature is scarce for what concerns this kind of robot-centered trust. Patacchiola and Cangelosi (2016) proposed a probabilistic model which unifies trust and ToM to be used in a simulation of Vanderbilt's experiment about children's trust willingness (Vanderbilt et al., 2011). This model has been subsequently expanded into a cognitive architecture for a humanoid robot (Vinanzi et al., 2019b) enhanced with an episodic memory system. The latter is a subcategory of the long-term declarative memory that stores memories about temporally dated episodes or events and temporal-spatial relations among them (Tulving, 1972). This feature is relevant because the positive influence of one's personal history on the cognitive capabilities has been proven other than for the biological brain also for artificial agents (De Castro and Gudwin, 2010; Jockel et al., 2008). Episodic memory is also the key to reproduce the “trust vs mistrust” stage theorized by Erikson (1993) in a developmental cognitive system.

In this paper, we present an integration of our previous studies on artificial intention reading (Vinanzi et al., 2019a, 2020) and trust estimation (Vinanzi et al., 2019b) to create a collaborative intelligent embodied agent able to direct its efforts in providing assistance in a shared activity with a human partner. Through the use of this computational model, we aim at demonstrating the positive influence of trust on the synergistic efforts of the two agents. Given this premise, our main contribution comes in the form of the novel cognitive artificial architecture for human-robot collaboration shown in Figure 2, capable to perform both intention reading and trust estimations on human partners. To achieve this, we have made use of a set of state-of-the-art techniques ranging from unsupervised machine learning methodologies to probabilistic modeling. We have validated this architecture through a set of simulated HRI experiments involving several humans and a robot collaborating in a block placing game. The results we collected demonstrate that the pairing of these two cognitive skills can greatly enhance the outcome of the joint action by providing the robot with some decision-making parameters that are used to fine-tune the assistive behavior.

## Results

### Experiments

Many of the considerations made throughout this section refer directly to the methodology involved in this line of research. For this reason, we invite the reader to consult the Transparent methods section of the Supplemental information to gain a better insight on the experiments that are described below.

Having already validated the performance of our intention reading (Vinanzi et al., 2020) and trust (Vinanzi et al., 2019b) models in our previous publications, the aim of our current experiment is to verify our hypothesis on the positive influence of trust mechanisms on the overall collaborative performance. For this reason, we are going to use the same experimental setup of our previous investigation on robotic mind reading and compare the results achieved from our new, integrated architecture (referred as trust architecture, or TA) with the baseline obtained from our previous intention reading model (Vinanzi et al., 2020) which we will hereafter be referring to as the no trust architecture, or NTA.

**Figure 3:**
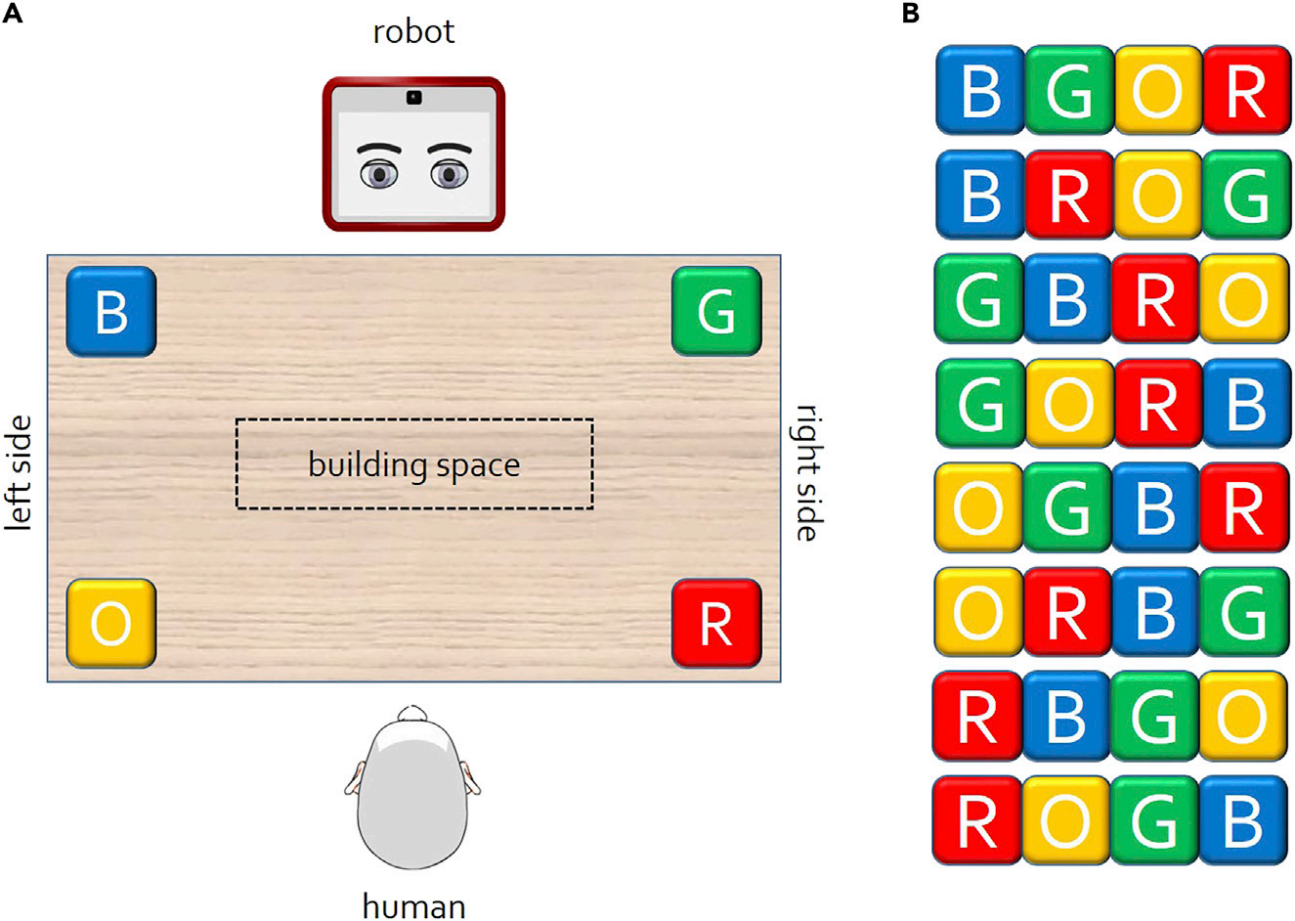
Experimental setup for the block building game (A) Schematic of the playing table, depicting the position of the 4 colored blocks: blue (B), orange (O), red (R), and green (G). (B) The 8 admissible block sequences obtained by picking blocks alternatively from each side. These sequences are the goals for this scenario.

The experimental setup is shown in Figure 3A. A Sawyer robot and a human are facing each other on the two sides of a table. Four different colored blocks are positioned on the corners of the playing area; anticlockwise from the top left they are blue (B), orange (O), red (R), and green (G). The central area of the table is denoted as the building space.

The aim of the game is to use the 4 available blocks to form a line, following a simple rule only known by the demonstrator: the blocks must be chosen one by one from a different side of the table (left or right). The 8 legal combinations of blocks are reported in Figure 3B and each of them forms a goal for our intention reading purposes. During the training phase, the human will demonstrate each goal once and the robot will learn to associate the demonstrator's body posture and eye gaze direction to their intentions. Additionally, the robot will always naively trust its teacher, while the beliefs regarding other subsequent partners will be generated using episodic memory. For more details on our adopted methodology, please refer to the Transparent methods section of the Supplemental information.

During the execution phase, the robot will follow the workflow described in the Transparent methods (Section S1.3). In our setting, a total output represents a full line of 4 colored blocks, while the partial output (PO) is the sequence of cubes that the human has arranged before the artificial agent was able to perform intention reading. If the human is trusted or the PO is valid, the robot will collect the next predicted blocks and hand them over to him or her. If not, the robot will position the blocks itself on the building area in what it considers to be the correct order, attempting to rectify the errors that have been committed. In the latter case, the robot will also offer an explanation of why it thinks the PO is invalid (in our experimental setting, this happens when two blocks from the same side of the table are placed one next to another).

In the scope of this experiment, an interaction will be considered successful if its outcome is a structure that follows the game's rules, in other words one of those listed in Figure 3B. This is true even if the true goal was not the one predicted by the robot: this is because we do not wish to measure the performance of the intention reading model (which has already been quantified) but rather we want to evaluate the collaborative effort itself. From here on, we will define a “positive” interaction one in which the human correctly achieves a valid goal and a “negative” one where he or she takes an unsuccessful course of action. The human might violate the rules more or less intentionally, but for our purposes, we consider both these cases as a failure that will lead to a decrease of their trust level.

To verify and measure the trust model's impact on the collaborative effort driven by the intention reading architecture, we have conducted a batch of simulated experiments (The use of virtual agents in a simulated environment is a COVID-19 lockdown contingency choice) using a virtual robot which has been modeled in accordance to the empirical data collected during our latest experiment on intention reading (Vinanzi et al., 2020).

After training the robot, we let it interact with a set of simulated humans which possess different behavior patterns. It is important to note that in most of these experiments we do not make an explicit use of episodic memory. This is because, having only familiarized with the demonstrator, the robot would generate a fully trustful network for the novel informant because it will be sampling episodes from a batch of positive memories. This mean that, for the purpose of the simulated experiment, we can simply assume that the robot will naively trust its new informant. Thereafter, we continue not using the memory system because we do not want our results to depend on the order in which the robot has experienced the users, rather we want to study how each robot would respond to each user independently. For completeness, one of our simulated humans is initialized with a distrustful BN to simulate the effects of the episodic memory.

We have divided the simulated humans in two groups. The first one involves the “deterministic” agents, which have a fixed behavioral pattern, as follows:

• $H_{1}$: always negative;
• $H_{2}$: 50% positive, then 50% negative;
• $H_{3}$: 50% negative, then 50% positive;

The second group categorizes the “stochastic” agents: the latter possess different success-to-failure ratios, but the order of their actions is randomized and not fixed. In particular, we have the following:

• $H_{4}$: 50% success rate;
• $H_{5}$: 80% success rate;
• $H_{6}$: 20% success rate;
• $H_{7}$: 80% success rate, but initialized with a distrustful BN;

The deterministic humans have been tested through a batch of 100 iterations each. For the stochastic ones, we have performed 10 random initializations, and for each of them, we have executed 100 interactions with the simulated robot. The only exception is $H_{4}$, for which we performed 20 random initializations due to its high variance. During each test, we have recorded the success rate and the opinion value, both of which are described in the following section.

### Evaluation metrics

#### Success rate

Given a human partner $H_{i}$, we define the success rate *S* as follows:

(Equation 1) $$S\left(H_{i}\right)=\frac{\text{successful goals}}{\text{total interactions}}\in \left[0,1\right]$$

We wish to formulate a comparison between the integrated cognitive architecture and the NTA. To do so, we refer to the success rate calculated on the latter as $S^{⋆}\left(H_{i}\right)$ and we formalize the difference between the two systems as follows:

(Equation 2) $$\Delta S\left(H_{i}\right)=S\left(H_{i}\right)-S^{⋆}\left(H_{i}\right)$$

Positive values of $\text{Δ}S\left(H_{i}\right)$ will denote a more performative collaboration obtained by our current architecture over the NTA and vice versa.

### Artificial opinion

We define a quantitative index which reflects the willingness of the robot to change its opinion about a partner. For a partner $H_{i}$ at a certain time step *t*, this artificial opinion is calculated as follows:

(Equation 3) $$O\left(H_{i},t\right)=\frac{n_{p}-n_{n}}{n_{p}+n_{n}}\in \left[-1,1\right]$$

where $n_{p}$ and $n_{n}$ indicate, respectively, the number of positive and negative episodes experienced by the robot with partner $H_{i}$ at time *t*. We will sometimes use a more simple notation, where we indicate the opinion of a robot toward a generic partner at a certain timestep simply as O(H).

When the robot trusts the person, that is, when $P_{X_{I}}\left(a\right)>P_{X_{I}}\left(b\right)$, it is also true that O(H)≥0 and vice versa, when the BN is distrustful toward them O(H)<0. The choice of having the robot to trust when $P_{X_{I}}\left(a\right)=P_{X_{I}}\left(b\right)$ and O(H)=0 is made by design since we wish the robot to act more friendly toward its users, giving them the benefit of doubt. The closest O(H) is to 0, the easier it will be for the agent to flip its trust and vice versa, and the more this value tends toward the extremes, the less inclined the robot will be to alter its belief. Of course, O(H)=±1 indicates a very strong opinion and it is possible only when the agent has experienced solely positive or negative episodes with that specific user.

### Success rates

In our last experiment on intention reading (Vinanzi et al., 2020), we have considered partners which always act toward one of the correct goals. This means that for a hypothetical human $H_{0}$ acting always positively, $S\left(H_{0}\right)=S^{⋆}\left(H_{0}\right)=1.0$ despite the fact that the empirical results we collected during that experiment indicate that the robot succeeds 80% of the time: this is because in the current investigation, we are not testing the intention reading capabilities, which enable the collaboration in the first place, rather we want to analyze the effect of a trust mechanism to correct partners who are not capable or willing to achieve a valid goal.

**Figure 4:**
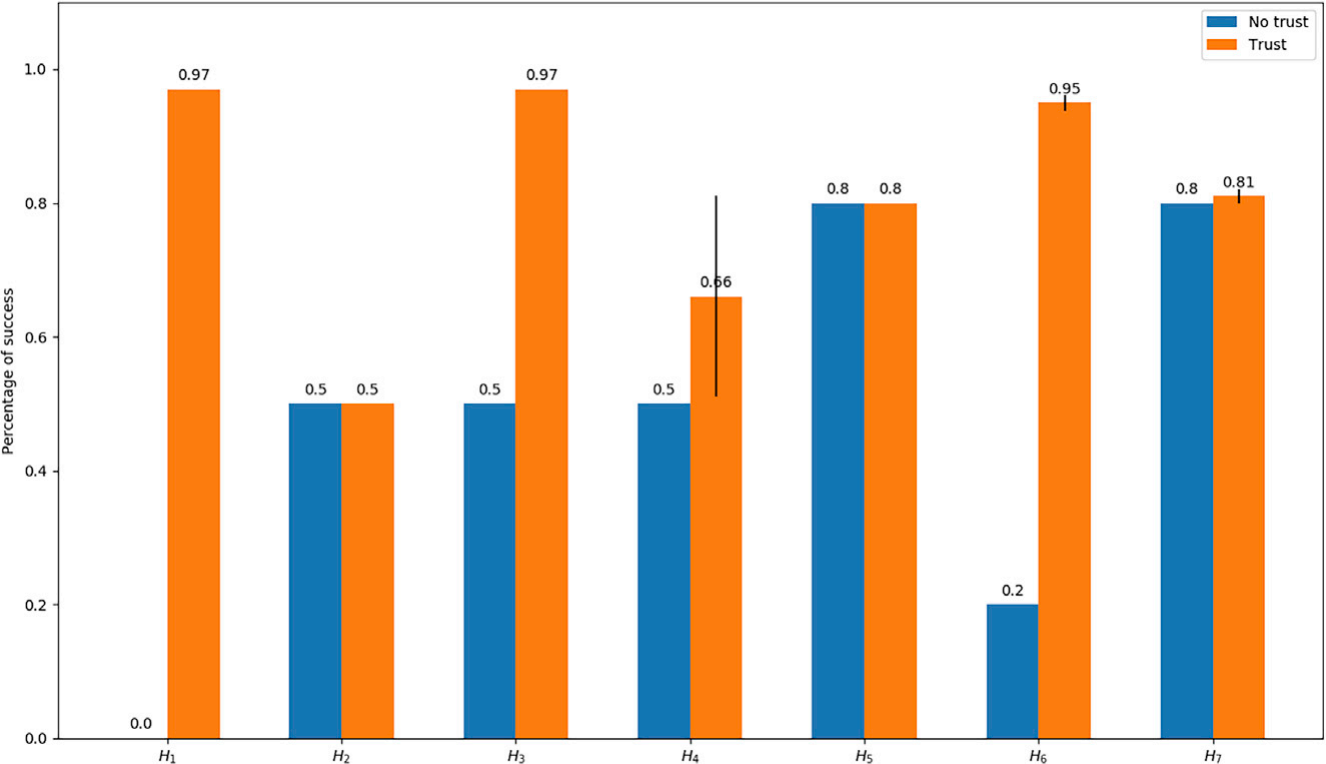
Comparison of the collaboration success rates with and without the trust model for each of the simulated informants

However, if we start considering humans which can (more or less intentionally) fail the task, the NTA's success rate drops drastically as it does not possess the ability to adopt any corrective actions. In this case, each action failed by the human will result in a failed collaboration. Figure 4 shows a comparison between the success rates of the two architectures measured on the 7 simulated humans. $H_{1}$ always fails the task so $S^{⋆}\left(H_{1}\right)=0$, while the trust-enabled model is able to score $S\left(H_{1}\right)=0.97$ with a significant increase of $\Delta S\left(H_{1}\right)=0.97$.

Both $H_{2}$ and $H_{3}$ provide a mixed scenario in which the behavior of the simulated human is quite regular by being respectively positive and negative for half of the time, in inverse order. In both these cases, the NTA could only score $S^{⋆}\left(H_{2}\right)=S^{⋆}\left(H_{3}\right)=0.5$. The trust mechanism did not prove itself of much use for $H_{2}$ since the robot builds up a strong trust for the user and is not able to change its mind in time to correct the new behavior: as we will see in the next section, this is because the agent should be observing at least $n_{p}+n_{n}+1$ negative cases to completely change its mind about the informant, which is not possible in this 50-50 split case initialized with positive episodes. In summary, $S\left(H_{2}\right)=0.5$ and $\Delta S\left(H_{2}\right)=0$, in other words the performance is the same as the one obtained through NTA. $H_{3}$ behaves similarly: not having enough time to change its mind, the robot continues to distrust the human nearly until the end. The difference is that in this condition the robot maintains a strict supervision on the interactions, leading to $S\left(H_{2}\right)=0.97$ with an increase of $\Delta S\left(H_{3}\right)=0.47$.

To better evaluate the stochastic humans, we have recorded the success rates achieved through the batches of random initializations and we have calculated the mean μ and the standard deviation σ. The success rates reported in Figure 4 for these simulated people represent the mean score, supplied with error bars representing σ. These values are also recorded in Table 1 for better visualization.

**Table 1:** Mean and standard deviation of the success rates calculated on the interactions performed by the stochastic simulated humans

Partner	Mean (μ)	Standard deviation (σ)	Initializations
$H_{4}$	0.66	0.15	20
$H_{5}$	0.8	0.0	10
$H_{6}$	0.95	0.01	10
$H_{7}$	0.81	0.01	10

$H_{4}$ is the agent who achieved the highest σ, that is because its behavior is the most unpredictable. This is explainable by considering what this behavioral pattern represents: with 50 positive and 50 negative episodes with randomized order of appearances, the trust levels can fluctuate significantly. This is also the reason behind our decision to execute double the number of trials with this simulated human. In this case, the NTA would have achieved $S^{⋆}\left(H_{5}\right)=0.5$, but the trust-enabled architecture is able to score $S\left(H_{2}\right)=0.66$, with σ=0.15, achieving on average $\Delta S\left(H_{4}\right)=0.16$. The performance of the TA has a theoretical lower bound equal to the one obtained by the NTA and in fact we have registered scores per batch not lower than 0.5, up to a maximum of 0.93. We can conclude that a success rate of 50% is a critical point of uncertainty in which the human's behavior is too variable for the robot to adapt efficiently. As we will see shortly, above this value, the human becomes more skilled and the value of trust-based corrective mechanisms gradually fades away and vice versa, lower success rates benefit more from the TA.

$H_{5}$ is a fairly expert human who succeeds 80% of the time, which means that $S^{⋆}\left(H_{5}\right)=0.5$. The robot builds a very solid trust toward this partner, at the point that the 20 failures are, in our experiments, sufficiently sparse in the set of 100 interactions to never make the trust flip to negative. The latter is of course theoretically possible, but they should appear clustered at the beginning of the batch to make that occur. This means that the robot never looses trust toward this confident human but that also those 20% failures are not being captured, hence $S\left(H_{5}\right)=0.8$, $\Delta S\left(H_{5}\right)=0$, and σ=0. This result is quite important because, as we mentioned previously, it demonstrates that the overall effectiveness of trust evaluations on the collaboration is inversely proportional to the skill of the partner.

The behavior of $H_{6}$ is quite the opposite of $H_{5}$, succeeding only 20% of the times. In this case, $S^{⋆}\left(H_{6}\right)=0.2$, but the full architecture was quickly able to detect the negative attitude of this simulated human and it promptly started distrusting them, achieving $S\left(H_{6}\right)=0.95$ with σ=0.01, leading to an average $\Delta S\left(H_{6}\right)=75$.

$H_{7}$ has the same behavioral pattern than $H_{5}$, which is an 80% success rate, but the robot facing him or her is not initialized with a trusting BN but rather with a naively distrustful network. This is meant to test the effects of the episodic memory on the performance of the architecture. As we can see from Figure 4, we achieve a similar result as $H_{5}$, just slightly better because the robot will tend to not trust them and take over the task until it is persuaded about their skill. The mean result for this scenario is $S\left(H_{7}\right)=0.81$, with σ=0.01 and $\Delta S\left(H_{6}\right)=0.1$. What this result stands for is the fact that the episodic memory has only a local effect on the robot's behavior, which is tuned on the long term through real interactions which take over its initial prejudice.

Overall, the experiments showed an average success rate increase equal to the following:

(Equation 4) $$\frac{1}{7}\sum_{i=1}^{7}\Delta S\left(H_{i}\right)=0.33$$

Thus confirming the positive impact of trust estimation in support of intention reading during collaborative HRI.

### Trust dynamics

During the simulated interactions, we have recorded the opinion value for each of the human partners. By a design choice, the robot is initialized with a trustful BN built from 4 positive episodes. This network yields an initial opinion O(H,0)=0.4. After that, we recorded O(H,t) for t∈{1,100} and we reported them in a set of graphs.

**Figure 5:**
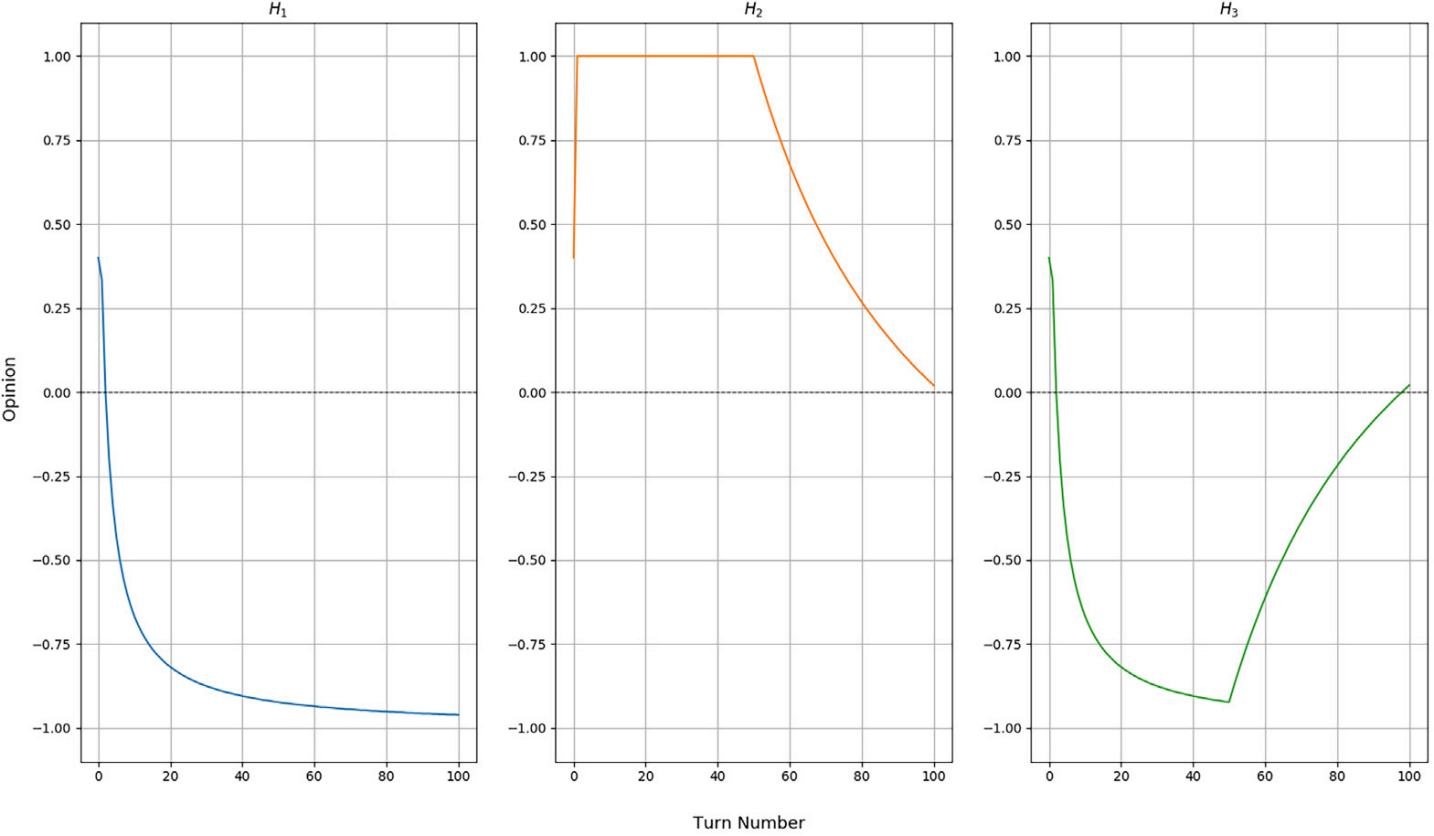
Variation of the opinion value at each turn of interaction for the 3 deterministic simulated informants ($H_{1}$, $H_{2}$, and $H_{3}$), initialized with a trusting BN When O(H) becomes less than 0, the robot starts distrusting the informant and taking more control on the task.

Figure 5 shows the dynamics of the robot's opinion through the various interactions for the deterministic humans. $H_{1}$ always acts incorrectly, but the network is initially willing to trust them. This changes very quickly since we can observe the opinion dropping to 0 after only a few negative episodes and then decreasing close to the lower limits. This value never actually reaches the minimum value of −1 because this would only be possible if the robot had experienced 100% negative episodes, which is not the case due to how its BN was initialized. In any case, we can see how the opinion of the robot stays low, meaning that the human will have to put a lot of effort to regain its trust.

$H_{2}$ behaves 50 times positively and, subsequently, 50 times negatively. During the first half of the interactions, the opinion raises to its maximum since the robot has only experienced successful interactions with that person. From turn 51 onward, the human starts failing the block building game and the opinion slowly decreases but it is not able to flip. This is because, by the end of the session, the robot possesses 54 positive and 50 negative episodes in its memory, meaning that it has not enough time to change its mind (other 4 negative episodes will bring the opinion to 0 and another one after that will flip the trust).

$H_{3}$ acts in the opposite way as for the previous simulated agent. The trust quickly drops in the distrusting side of the graph and slowly rises after turn 50. In contrast with $H_{2}$, this human is able to flip the trust back to positive by the end of the session because of the way it was initialized. If the BN was originally set to distrust, these two graphs would result inverted.

As previously mentioned, the random nature of the stochastic humans required several batches of iterations, performed with different random initializations, to fully understand the behavior of each agent.

**Figure 6:**
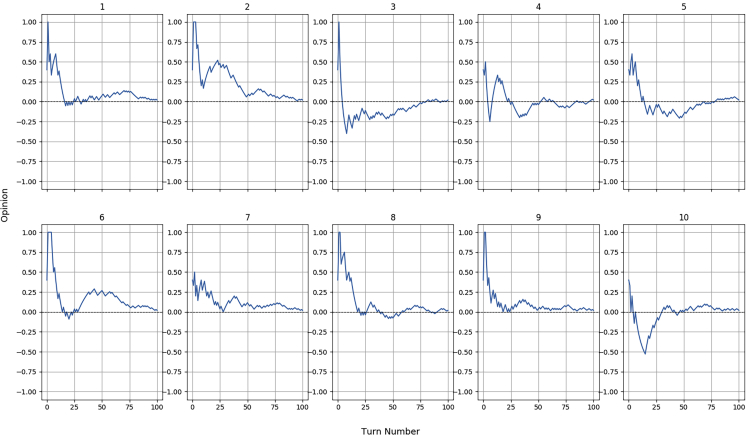
Opinion dynamics for the stochastic human $H_{4}$ (50% success rate) during its first 10 iterations

Figure 6 reports the dynamics of the robot's opinion during 10 out of the 20 iterations performed for $H_{4}$, which is the simulated agent with a success rate of 50%. What is immediately noticeable from these graphs is that the opinion always converges around 0: this is an expected result since this value is the midpoint in the scale, representing partners with mixed, indecisive behaviors. It is worth remembering that the robot will trust a human when O(H)≥0.

**Figure 7:**
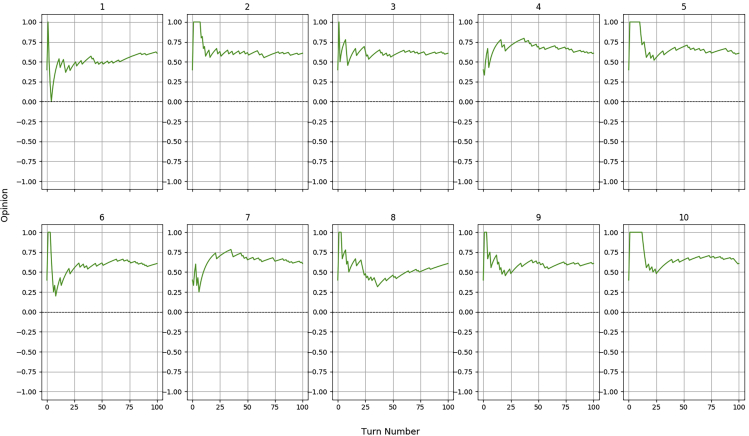
Opinion dynamics for the stochastic human $H_{5}$ (80% success rate) during 10 iterations

Regarding $H_{5}$, having a success rate of 80%, we expected the robot to terminate each iteration with a high opinion. This prevision was confirmed by the graphs reported in Figure 7, which show that the robot never fully changed its impression of the partner, in other words the 20 errors randomly scattered among the 100 interactions were not sufficient to flip the trust. The closest the robot got to distrust them happened in the first diagram, where a sequence of negative episodes were experienced right at the beginning, dropping the opinion to 0, which by our design still represents a trusting situation.

**Figure 8:**
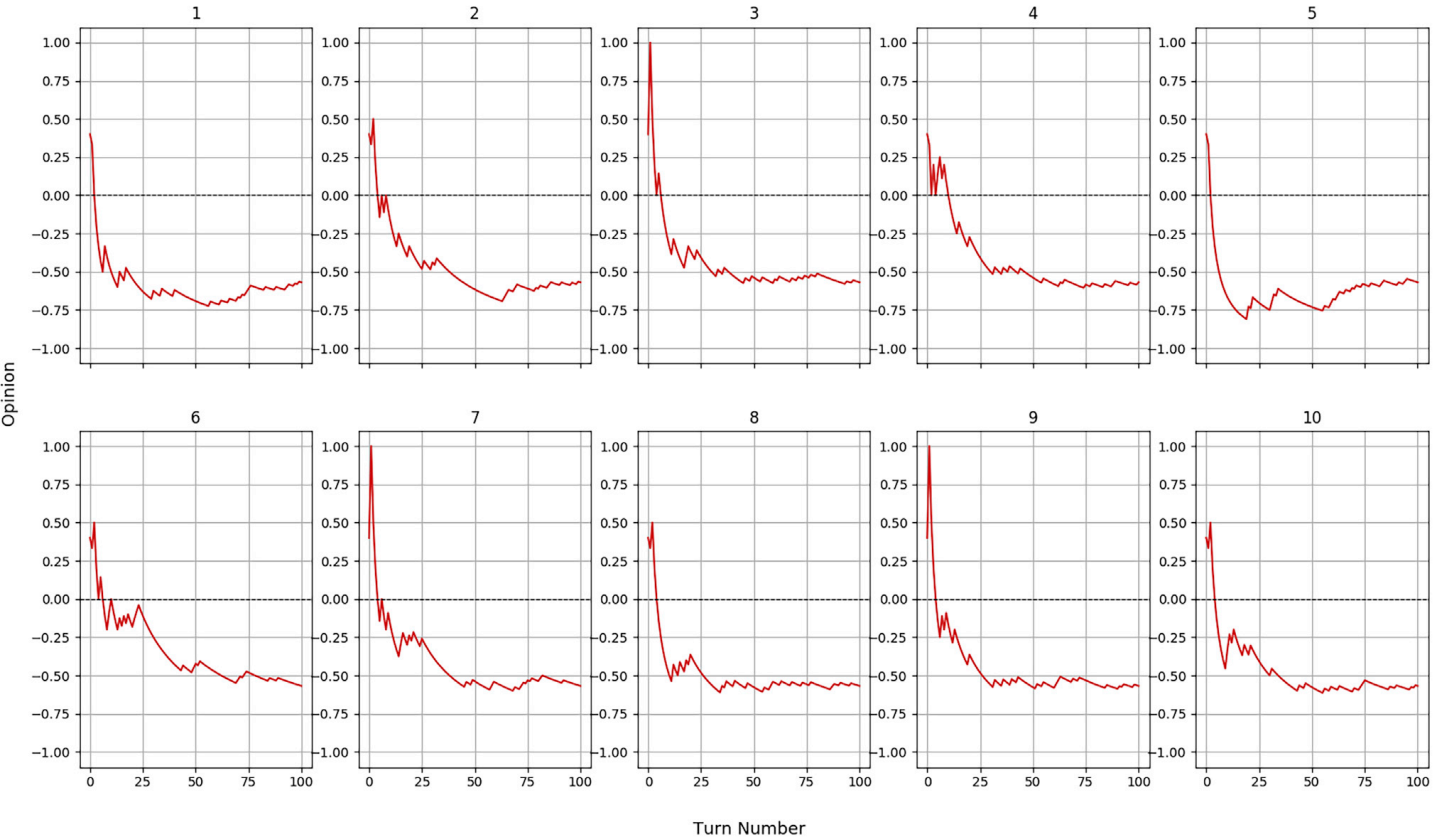
Opinion dynamics for the stochastic human $H_{6}$ (20% success rate) during 10 iterations

Similar considerations are valid for $H_{6}$, the virtual agent capable of a very low, 20% success rate. The 10 diagrams of Figure 8 differ mostly on the very first interactions, when a sequence of positive episodes may impact the limited memory of experiences of the robot and in fact some of the iterations have managed to achieve trust for some turns. Ultimately, the opinion always ends up settling on the lower side of the graph, in the distrust domain, which is what we expect from a human who consistently fails the majority of the tasks.

**Figure 9:**
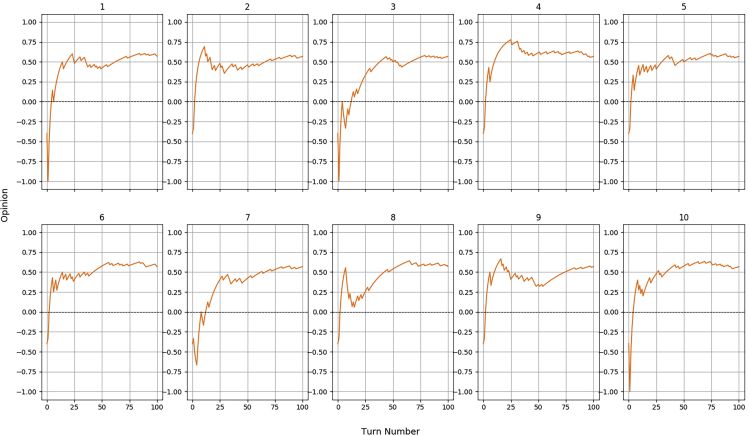
Opinion dynamics for the stochastic human $H_{7}$ (80% success rate, against a naively distrusting BN) during 10 iterations

All the previous simulations have been executed on a simulated robot initialized with a trustful BN, for the reasons we have explained in the preceding sections. We now wish to analyze what would happen if the network was created through episodic memory, that is, if it does not contain 4 positive episodes but a certain number of negative ones. For this reason, we have built $H_{7}$ with a BN composed of 4 negative episodes: this yields $O\left(H_{7},0\right)=-0.4$. Figure 9 shows the result of this experiment, which is comparable to the one performed for $H_{5}$ since these two simulated humans behave in the same way, with the only difference being the initial prejudice. Despite the variance in the early interactions, which can make the opinion oscillate quite widely, on the long run, the latter settles for similar values registered for $H_{5}$. This demonstrates that the episodic memory can create a local effect which influences strongly the early interactions of the robot with a person but that fades gradually once the actual experience takes over the initial prejudice. This is exactly how the episodic memory system was intended to operate. Having tested the two types of BN that can be generated by the episodic memory system (completely polarized toward trust or distrust), we do not feel the need to investigate the cases which lie in between: these will produce similar, but more mitigated, effects than the ones we have observed.

## Discussion

Collaboration between people has been, through history, the key to obtain the grand achievements of the human species. In a future world where humans and robots will be living closely, we want to be able to collaborate with them too. With this purpose in mind, we state that a true collaborative robot able to operate in human-sized environments must possess the same cognitive skills that drive our own social life. In this paper, we defined collaborative intelligence as the mutual interaction between intention reading and trust estimation, two mental abilities toward which humans are biologically oriented. The former allows an agent to understand the actions and goals of other agents acting around it, thus providing clues and meaning to simple sensory perceptions, while the latter is essential to estimate the level of skill or knowledge of another agent so to formulate an appropriate plan. Following the developmental robotics principles, our cognitive architecture takes inspiration from scientific findings in human cognition, and both the intention reading and trust models are designed according to the current psychological literature. We have developed a cognitive system which is able to learn goals by demonstration in an unsupervised and probabilistic way and to estimate trust using an artificial ToM. We have applied this architecture to a block building game where a robot is engaged with several humans to pick and place some colored cubes from a table to form constructions that obey to certain patterns.

Overall, we can conclude that the synergistic combination of intention reading and trust leads to better results than the ones obtainable by just predicting the human's goal. The experiments that we conducted have shown that the complementary use of both these cognitive skills enhances the collaborative performance, making the robot act as a better teammate. This confirms our initial hypothesis, which is that collaborative intelligence is enabled by the ability to read another agent's intention and is fostered by the capacity to correctly estimate the trustworthiness of the other party. The robot's ability to take control of the task whenever the partner demonstrates a lack of skill results in a significant increase in the success of the joint task.

Both the intention reading and the trust models offer directions in which to orient future investigations. The former, for example, could benefit from the addition of hierarchical goals, i.e., goals composed by multiple sub-goals (for example, uncapping a bottle might be one step to achieve the “drink” goal). Another possible study could explore the use of more social clues and the investigation of their order of application within Feature-Space Split Clustering, the multi-modal clustering algorithm which we use within the intention reading module, described in detail in the Supplemental information. Both these components will be revised in the near future to apply them to multi-agent systems: ensembles of heterogeneous agents, each of which has the ability to contribute to a greater problem-solving network. In this kind of scenario, it would be possible to take into account the contemporary influence of two or more agents, similarly to what has been done by Butterfield et al. (2009). This, of course, would also imply the adaptation of this cognitive architecture to collaborate not only with humans but also with other artificial agents.

Another future direction could involve the use of this architecture within a more continuous representation of trust, where a partner possesses a degree of trustworthiness as opposed to a binary state. Having access to a more refined representation could provide further benefits for the robot: for example, this could translate in a continuous definition of the collaboration process, where the agent might decide to take over only a subset of the actions based on their complexity.

## Limitations of the study

Due to the inability to access appropriate research facilities due to COVID-19 lockdown in the United Kingdom, it was not possible to perform experiments on the physical robot, which were instead replaced by simulations. By providing the virtual robot the same empirical error rates obtained during the foundational experiments, which have all been executed in the real world, we have tried to minimize any approximation errors between the simulated and the real interactions.

## Resource availability

### Lead contact

Further information and requests for resources should be directed to and will be fulfilled by the lead contact, Samuele Vinanzi (samuele.vinanzi@manchester.ac.uk).

### Material availability

This study did not generate new unique materials.

### Data and code availability

The data and code in this manuscript are available at github.com/samvinanzi/DeCIFER.

## Methods

All methods can be found in the accompanying Transparent methods supplemental file.

## Supplemental Information

Document S1. Transparent methods and Figures S1–S7
