# Supplementary material for: The collaborative mind: intention reading and trust in human-robot interaction
Source: iScience. 2021 Feb 1;24(2):102130. doi: 10.1016/j.isci.2021.102130 (PMC7890414; doi:10.1016/j.isci.2021.102130)
Supplement: Document S1. Transparent methods and Figures S1–S7 [file mmc1.pdf]

iScience, Volume *24*

Supplemental Information

The collaborative mind: intention reading and trust in human-robot interaction

Samuele Vinanzi, Angelo Cangelosi, and Christian Goerick

1 Transparent Methods

The aim of our research is to develop a comprehensive cognitive architecture that encompasses both intention reading and trust abilities for a humanoid robot engaged in HRI. To do so, we are going to build on the foundations of our previous models (Vinanzi et al., 2020, Vinanzi et al., 2019) and build an integration that, following the schematic presented in Figure 1, will allow a robot to act collaboratively towards a human partner. This architecture will be used in a scenario in which the robot will have to infer the goal of its partner by the observation of their social cues and subsequently perform decision-making to formulate an action plan that will try to optimize the chances of successfully achieving the intended objective.

For the design of both the artificial intention reading and trust capabilities, we made use of the developmental robotics approach. Cangelosi et al. (2015) defined this subject as "the approach to the design of behavioral and cognitive capabilities in artificial agents that takes direct inspiration from the developmental principles and mechanisms observed in the natural cognitive systems of children". In other words, our computational models are inspired by scientific findings in human cognition.

1.1 Intention Reading

1.1.1 Motivation

In the pursuit of the developmental robotics approach, the intention reading model lacks a pre-existing plan library, rather it follows the psychological theories which state that this cognitive ability is learned by experience (Malle et al., 2001). Furthermore, it follows the principles theorized by Tomasello et al. (2005) which state that the intention decoding task is divided in a low-level action understanding based on social cues and a high-level goal prediction. The lack of a hand-crafted plan library means that the robot will be able to learn goals in a more flexible and scalable way, while the use of unsupervised an probabilistic models, rather than supervised ones such as neural networks, makes the robot learn on the fly with no need for big datasets or long traning times, making this architecture lightweight on a computational point of view.

An overview of the intention reading architecture is shown in Figure 1. Additional details about this section can be found in our previous publications (Vinanzi et al., 2019, 2020).

1.1.2 Low-level social cue clustering and action representation

The low-level module of the intention reading architecture tries to encode temporal sequences of human configurations, expressed as sets of social cues, into a more compact representation that will be used to recognize actions. The main idea is to observe the human acting to achieve the goals, then cluster the set of their social cues and analyze how their actions unravel through these clusters to form an encoding that will be used by the high-level goal prediction module.

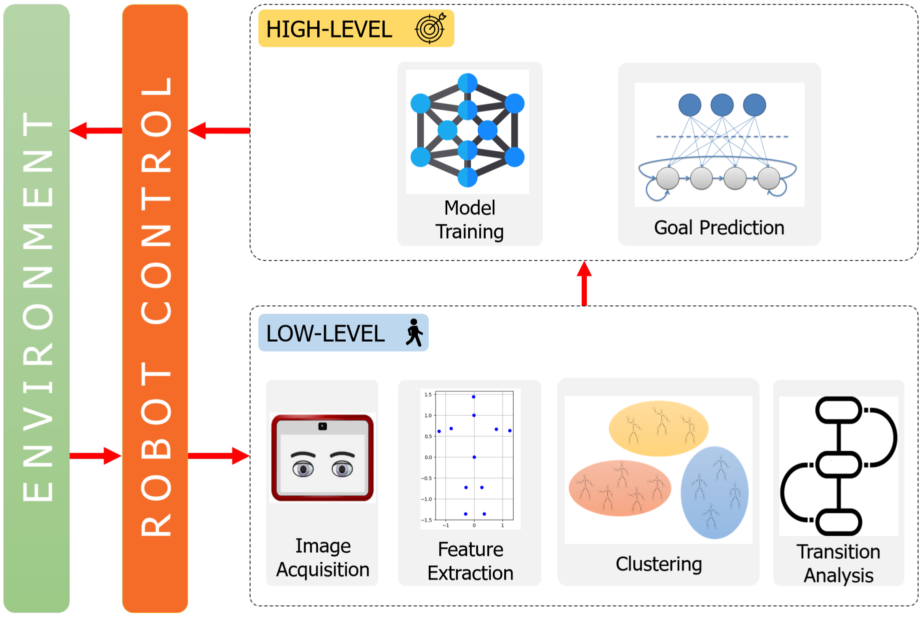

Supplemental Figure 1: Overview of the intention reading architecture. The low-level extracts social data from the optical stream, forms clusters and uses them to represent actions as transitions through clusters. The high-level uses this encoding to probabilistically infer the pursued goal. The robot control manages sensors and actuators. Related to Figure 2.

Following the psychological literature (Tomasello et al., 2005), we chose to employ body posture and eye gaze direction as the features that the robot is going to observe from the human.

We collect postural information by generating skeleton data through the use of a pre-trained deep convolutional neural network architecture named OpenPose (Cao et al., 2016), specialized in real-time multi-person 2D pose estimation. This neural network receives in input the images from the robot's eye camera and outputs a 18x2 feature vector representing the detected skeleton keypoints as 18 joints expressed in 2D spatial coordinates, as reported in Figure 2a. In order to optimize memory and speed requirements and to comply with recent findings which state that classification tasks achieve better results with a reduced set of joints (Manzi et al., 2017), we operate a keypoint reduction to diminish the volume of data required for each skeleton. To do so, we discard the keypoints corresponding to the eyes, ears and shoulders, whilst calculating a new torso keypoint as a median between the two hips: doing so, we obtain a more compact 11x2 representation shown in Figure 2b.

The skeletons generated by this procedure cannot be used directly for classification purposes, as they are dependent on the position and size of the subject.

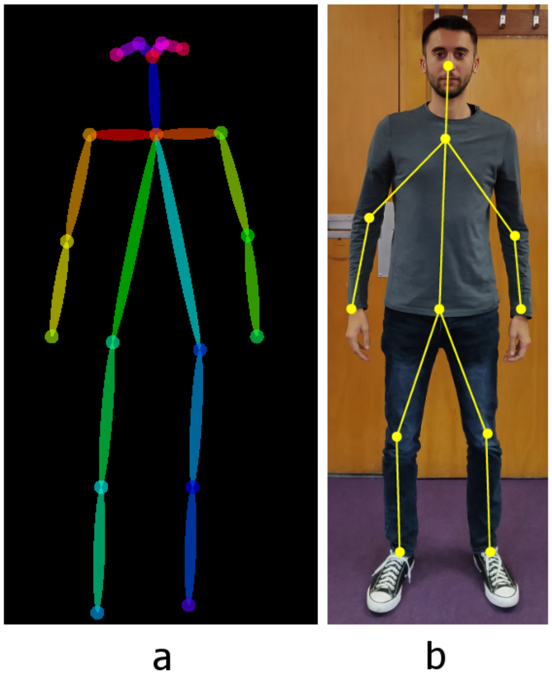

Supplemental Figure 2: A comparison between the skeletal keypoints extracted from the camera image (a) and the reduced keypoint set computed by the system (b). Related to Figure 2.

To overcome this problem and obtain spacial invariance, we apply a normalization process introduced by Cippitelli et al. (2016). For a skeleton with n joints, the feature vector f is defined as:

$$f = [J_1, J_2, ..., J_n] \tag{1}$$

where J_i is a vector containing the normalized 2D coordinates of the ith keypoint:

$$J_i = \frac{J_i - J_0}{\|J_1 - J_0\|} \tag{2}$$

where J_0 and J_1 are, respectively, the neck and torso joint. The latter will be located on the origin of the cartesian space, so its components will all be zero. For this reason, it is removed from the feature vector, which at this point will have a dimension of 10x2: this corresponds to a 44.5% size reduction from to the original representation.

Another social cue that we collect from the robot's partner is gaze direction. We use Deepgaze (Patacchiola and Cangelosi, 2017), a convolutional neural

network specialized in head pose estimation, to retrieve a 3D vector representing estimated roll, pitch and yaw of the human for each image acquired by the robot. We chose to approximate gaze direction with head orientation to avoid some computational overheads that would impair the real-time computation of several frames per second. This has been proved to be an acceptable approximation (Jha and Busso, 2017).

We create action representations from the perceptual data through an unsupervised clustering procedure, using a novel algorithm that combines multiple sets of features in several increasingly refined stages, which we call Feature-Space Split Clustering (FSSC). This strategy is adopted because it is possible to distinguish complex and potentially ambiguous actions by increasing the granularity of the clustering operation, which means taking into account a multitude of social cues. The main idea behind FSSC is a multi-level clustering process that uses only a subset of the features at each level.

Consider a set of M training samples:

$$X = \left\{ x^{(1)}, x^{(2)}, \dots, x^{(M)} \right\} \tag{3}$$

Each sample can be seen as defined by N groups of features:

$$x^{(i)} = \left\{ f_1^{(i)}, f_2^{(i)}, \dots, f_N^{(i)} \right\} \tag{4}$$

Each group defines the feature-space f_n with $n \in [1, N]$ and contains data extracted from a different perceptual input. In our scenario we use $N = 2$ and for each image i we have that $f_1^{(i)}$ is a 20D vector containing the skeleton keypoints configuration and $f_2^{(i)}$ is a 3D vector that specifies the gaze direction.

FSSC works by implicitly computing a tree of depth $L = N$ whose nodes contain the refined clusters. The root node ($\ell = 0$) contains all the data samples and is considered as a single cluster, while nodes of each subsequent level $\ell > 0$ are the clusters obtained by clustering the samples belonging to the parent node in the feature-space f_ℓ. At each level, we perform Principal Component Analysis (PCA) dimensionality reduction to project the data in a 2D space to avoid the curse of dimensionality (Bellman, 2013). We do so also because clustering relies on euclidean distance as a metric, but in high dimensional spaces the concept of distance becomes less precise, since it tends to converge. Finally, we chose X-Means (Pelleg et al., 2000) as the internal clustering method, which is a variation on the traditional K-Means algorithm that overcomes its principal limitation: the need to manually specify the parameter K that defines the desired number of clusters. The model selects the optimal one by performing model selection among a finite set of models through the optimization of the Bayesian Information Criterion. Algorithm 1 describes this computation.

Given the hierarchical and nonlinear nature of this algorithm, we can't perform classification (intended as the association of a new data sample to one of the existing clusters) through a simple Euclidean distance search for the closest centroid. Instead, the procedure described in Algorithm 2 must be adopted.

Algorithm 1: Feature-Space Split Clustering (FSSC)

Input: training samples X; number of feature sets N
Output: A tree of clusters
$tree \leftarrow \{\}$
Initialize the root node with all the samples X
for $\ell \leftarrow 0$ **to** N **do**
  foreach *cluster of level* ℓ **do**
    $x \leftarrow$ samples belonging to *cluster*
    $f \leftarrow f^{(x)}_{\ell+1}$
    $f' \leftarrow$ Dimensionality reduction on f
    $newClusters \leftarrow CLUSTERING(f')$
    Set $newClusters$ to level $\ell + 1$
    $tree \leftarrow tree \cup newClusters$
  end
end
return *tree*

The latter searches through the cluster tree, comparing the centroids in their respective feature-space coordinates until a leaf node is found.

It is important to note that, despite this approach splits the feature-spaces, in fact it does not decouple the multi-modal features extracted from the human, as they share a temporal dependence on the frame from which they were generated. In other words, the features never lose their alignment.

During its initial training, the agent will observe the actions of its human partner, record the image frames and extract all the relevant features. This assembled dataset will be used to create the clusters using the method described in Algorithm 1: each of them represents a group of similar but not identical postures. These will be used in the next stage, Transition Analysis, to create the low-level encoding: each action will be represented by the sequence of the cluster ids encountered during its performance. To obtain temporal invariance, we include in the encoding only transitions through different clusters, discarding the persistence in the same group: in this way, the representation of an action won't depend on the speed of its execution. The results of this analysis, plus a set of unique names for each goal which are generated automatically by the robot, are forwarded to the high-level module to train it as described in Section 1.1.3.

After being trained, the system will be able to perform intention reading: the agent will observe its human partner during the execution of the action, each of their physical configurations will be classified to one of the known clusters using the procedure described in Algorithm 2 and the discovered id will be forwarded to the high-level module for probabilistic inference.

The low-level intention reading process is sensitive to noise because it is learning each intention and its sequence of actions from a single training ex-

Algorithm 2: Cluster classification

Input: cluster tree T, testing sample s
Output: cluster to which s belongs
$parentNode \leftarrow$ root node of T
$\ell \leftarrow 1$
Loop
  $C \leftarrow$ descendants of $parentNode$ in T
  $cluster \leftarrow \min distance(f_\ell^{(s)}, c \in C)$
  if $cluster$ has descendants **then**
    $parentNode \leftarrow cluster$
    $\ell \leftarrow \ell + 1$
  else
    return $cluster$
  end
EndLoop

ample, which is in turn obtained from a non-deterministic and unsupervised process. To reduce this effect, we implement a post-processing computation that aims to ground this general architecture to our specific experiment by capturing the regularities of the data and assigning to each end position of our actions (i.e. the grasping position for each of the blocks) one of the cluster ids based on its statistical mode in the computed training dataset. Training actions are then eventually corrected by merging the two representations to avoid any errors in the training set.

1.1.3 High-level goal prediction

The high-level module is in charge of goal probabilistic inference from the observed actions. What we are trying to achieve is not action recognition but rather prediction, this means that only one part of the action will be known and observable. Our objective is to determine the intention based on as few observations as possible, so that the robot will be able to contribute to the task before it is over.

To achieve this, we have employed the BN shown in Figure 3. The top node denoted as I represents the intention of the human partner and its probability distribution is equal across all the possible goals. The bottom nodes marked as O_k with $k \in [1, K]$, where K is the maximum length of the encoded actions, represent the observations. The values of these nodes span in the range of the possible cluster ids identified by the low-level module. The conditional probability tables of the observation nodes are fitted from the training data provided by the low-level Transition Analysis (i.e. the action encoding associated to each goal name) using Maximum Likelihood Estimation (MLE) (Aldrich et al., 1997). We assume that the probability of each observation depends on the driving intention and by the precedent symbol encountered:

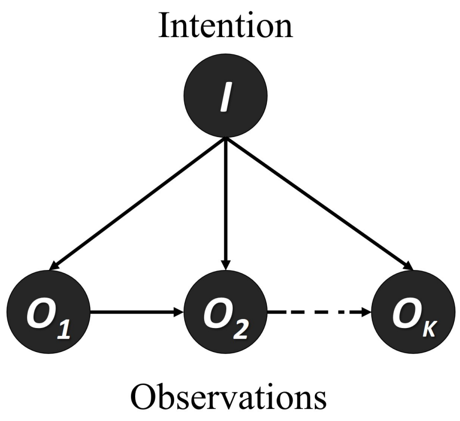

Supplemental Figure 3: The BN used for high-level probabilistic goal prediction. The top node represents the intention of the observed partner, whilst the bottom node symbolize the observations (the action encoding symbols produced by the low-level module). Related to Figure 2.

$$P(O_1 \mid I) \tag{5}$$

$$P(O_k \mid O_{k-1}, I)\colon k \in [2, K] \tag{6}$$

Once the probabilistic model is trained, it can be used for inference. During the execution phase, the robot will be observing the human and recording each cluster transition in real-time. The low-level module will forward these symbols to the high-level, which will treat them as sequential observations. Each time a new piece of evidence is added to the model, we use Pearl's Message-Passing algorithm (Lauritzen and Spiegelhalter, 1988) to calculate the marginal probability distribution for node I given the evidence. As soon as one of the goals is predicted with a probability greater than 0.5, it is sent forward in the processing chain to instantiate appropriate collaborative behavior. The value of this threshold was chosen in compliance to the time restrictions: we could choose to wait for a higher confidence, but this may slow the prediction time up to the point in which the robot's intervention in the joint task would become irrelevant.

1.1.4 Robot control

The robot control module deals with the direct interface between the cognitive architecture and the robotic platform, in this case a Sawyer: an industrial collaborative robot designed for object manipulation, equipped with a 7-DOF arm (Figure 4). In particular, it provides interaction with the ROS middleware to

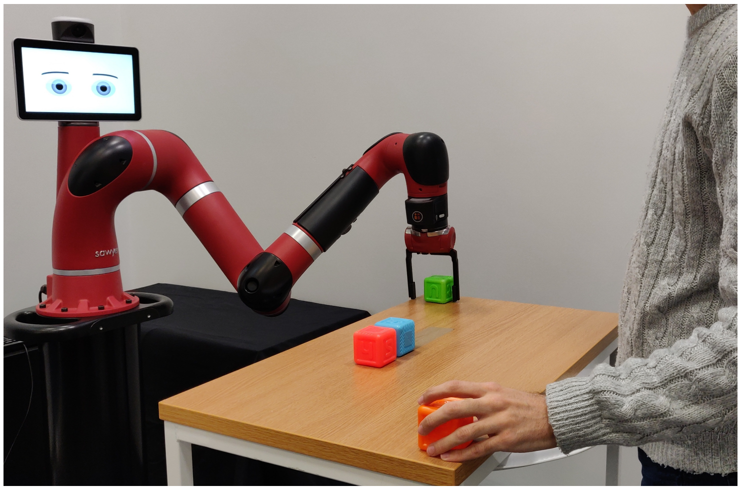

Supplemental Figure 4: The Sawyer robot which was simulated for the collaborative intelligence experiments involving an interactive block placing game. Related to Figure 2 and Figure 3.

control its sensors and actuators and perform vision, movement and grasping for the shared goal task.

1.2 Trust Estimation

1.2.1 Motivation

The trust model has been designed to be able to reproduce the psychology experiment on ToM maturity by Vanderbilt et al. (2011). In the latter, 90 preschool-age children equally divided in 3-, 4-, and 5-years-olds were exposed to a video in which an adult actor gave advice to another adult who was trying to locate a sticker hidden in one or two boxes. The informant could be either a helper or a tricker, suggesting respectively the correct or the wrong location. In the second phase of the experiment, a child would be involved in the game and would receive the same kind of suggestion by the informant. Based on the children's choices and on some meta-cognitive questions submitted to them, Vanderbilt theorized that only the 5-year-olds were able to differentiate the helpers from the trickers, therefore demonstrating to possess a mature ToM.

1.2.2 Bayesian approach in trust reasoning

In our previous research (Vinanzi et al., 2019) we developed a probabilistic model that could allow a robot to take part in the same sticker finding experiment and to act as a child with mature or immature ToM, learning to predict the beliefs and attitudes of the informants. The trust estimation architecture

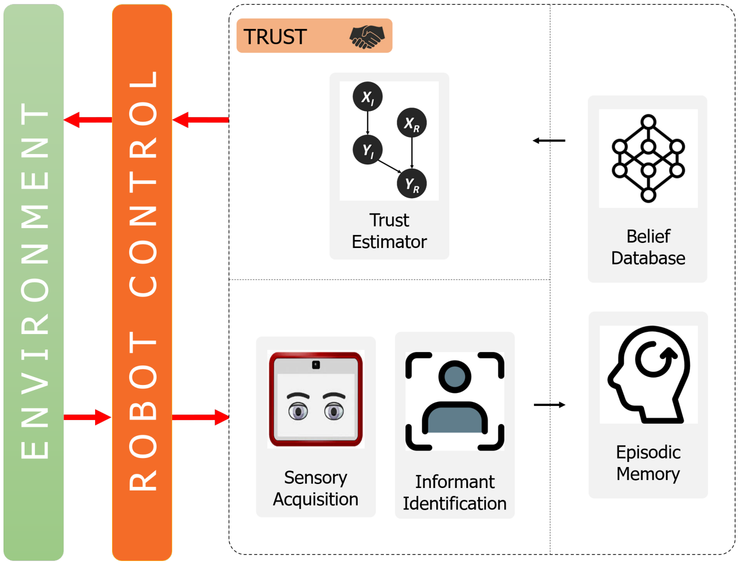

Supplemental Figure 5: Overview of the trust estimation architecture. The robot uses its sensors to identify each human and select their own BN or eventually generate a new one using episodic memory. The selected model is then used for inference. Related to Figure 2.

is reported in Figure 5. We employed a BN using discrete Boolean variables that assume two states: a and b, each corresponding to one of the two positions where the stickers can be located in the experiment. A graphical illustration of this BN can be observed in Figure 6: the two nodes X_R and Y_R represent respectively the beliefs and actions of the robot. The posterior distribution of the node Y_R allows the agent to choose the action to perform: that means searching for the sticker in position a or b. The connection between Y_I and Y_R represents the influence that the opinions of the informant have on the agent's action. The action of the agent is then a consequence of its own belief X_R and the informant action Y_I. Lastly, the estimation of X_I, the informant's belief, makes the agent able to effectively discriminate a trickery from a non-malevolent human error. The cognitive architecture we designed creates one of these BNs for each human it interacts with and uses it to predict their future behavior. Every partner is detected and recognized using Haar Cascade (Viola and Jones, 2001) and Local Binary Pattern Histogram (Ojala et al., 2002) on the robot's camera live stream.

For our current purposes, we intend to employ this model to check whether the human has the knowledge or skill to achieve a given goal: if this is not the case, the robot will have to perform corrective actions to ensure the success of the task. To do so, we have employed the same Bayesian network changing the meaning of its binary nodes: a will represent a correct goal whilst b will symbolize an incorrect goal. Following this convention, X_I and X_R will

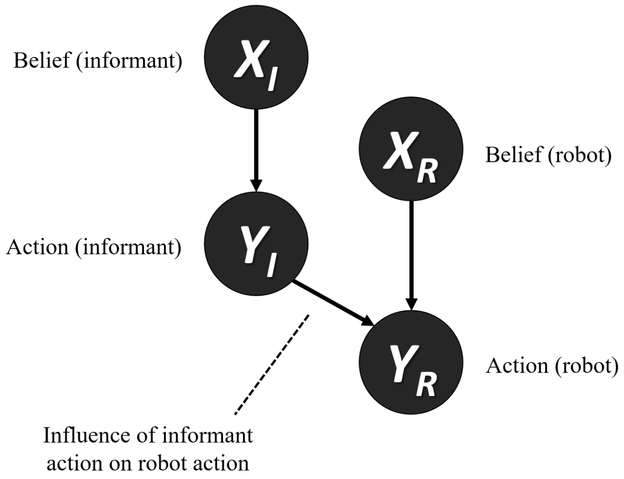

Supplemental Figure 6: The BN that models the relation between the robot and an informant. The agent generates a separate network for each user, with the same structure but different probability distribution. Related to Figure 2.

represent, respectively, knowledge of the informant and of the robot about the correct goals, Y_I will symbolize the choice of a correct or incorrect action by the informant and finally Y_R will depict whether the robot should adopt a trusting or a corrective action.

The trust model is built from episodes, which are data structures that encode interaction outcomes. Once the agent has collected a certain amount of episodes from an informant, it can generate a BN associated to him or her using MLE to determine the conditional probability tables of its nodes. For the root nodes X_I and X_R we calculate these probabilities as:

$$\begin{aligned} P_Y(a) &= \theta \\ P_Y(b) &= 1 - \theta \end{aligned} \tag{7}$$

Denoting N_a and N_b as the number of times the human demonstrates a or b, we can estimate θ as:

$$\hat{\theta} = \frac{N_a}{N_a + N_b} \tag{8}$$

For the nodes Y_I and Y_R, instead, we have to also take into consideration the influence of the parents.

Once a BN has been created for a certain user and its parameters have been learned from the interactions, it is possible to infer the posterior probability of the nodes given some observations. We calculate posterior distributions using Pearl's Message-Passing algorithm (Lauritzen and Spiegelhalter, 1988).

The outcome of each interaction is saved as a new episode that will modify the parameters of the probabilistic model. This means that while experiencing new interactions, the BN can acquire new statistical data and adapt its behavior over time, eventually switching between trust and distrust.

1.2.3 Episodic Memory

The power to use one's own past memories to take decisions in the present and future is an important ability that enhances the cognitive processes. In the original experimental design by Vanderbilt et al. (2011), the child (or, in our case, the robot) would familiarize with the partner before the real interaction. We make use of episodic memory to let the artificial agent be able to instantly interact even with unfamiliar people. On a technical level, the main problem is to generate on the fly a new BN with adequate parameters to use with that unknown person. These parameters will depend on the robot's personal character which, in turn, depends on the way it has been treated in the past: an agent which has often experience human failures would learn to be mistrustful and vice versa, as in the "trust vs mistrust" phase in child development (Erikson, 1993).

The design guidelines that we followed in the creation of our algorithm were the following: memories fade away with time, the details become blurred proportionally to the amount of memories possessed and, finally, shocking events such as surprises and betrayals should be more difficult to forget. Our algorithm draws inspiration from the particle filter technique widely used in mobile robot localization (Rekleitis, 2004). Whenever an unknown informant is met, this component generates on the fly a certain number of episodes to train a new BN.

We define the set of BNs memorized by the agent as:

$$S = [s_0, s_1, ..., s_n] \tag{9}$$

Where n is the number of humans known by the agent.

Each BN s_i was generated by a set of episodes, and these are going to be denoted as *replay datasets* for that BN:

$$E_{s_i} = [\varepsilon_0^{(s_i)}, \varepsilon_1^{(s_i)}, ..., \varepsilon_m^{(s_i)}] : s_i \in S \tag{10}$$

Where m is equal to the number of episodes of the replay dataset. So, in this notation $\varepsilon_j^{(s_i)}$ represents the j-th episode of the replay dataset that formed the BN s_i.

The equation we are about to introduce uses information theory to quantify the amount of information each specific episode represents. Our goal is to find how much this value differs from the total entropy of its replay dataset: a high difference means that the event is to be considered surprising and must be easier to recall than ordinary, unsurprising events. For example, if a person who is always been trustful suddenly tricks the agent, this betrayal will be remembered with a greater impact. At the same time, all of the memories are subject to a

progressive time degradation that tends to blur them with a timing dependent on their importance.

Formally, a real factor denoted as importance value v defined in the interval $[0, 1]$ is calculated for every episode $\varepsilon_j^{(s_i)}$ as the difference between the amount of information of the episode, $I(\varepsilon_j^{(s_i)})$, and the total entropy of its replay dataset, $H(E_{s_i})$, divided by the discrete temporal difference from the time when the memory was formed.

$$
\begin{aligned}
v(\varepsilon_j^{(s_i)}) &= \frac{\mid I(\varepsilon_j^{(s_i)}) - H(E_{s_i}) \mid}{\Delta t + 1} \\
&= \frac{\mid -\log_2 P(\varepsilon_j^{(s_i)}) + \sum_{\varepsilon \in E_{s_i}} P(\varepsilon) \log_2 P(\varepsilon) \mid}{t_{present} - t_{\varepsilon_j^{(s_i)}} + 1}
\end{aligned}
\tag{11}
$$

Equation 11 is used to weight every episode from each replay dataset in the agent's memory in order to perform a systematic resampling (Douc and Cappé, 2005) to pick the new episodes that will form the replay dataset for the new BN we intend to create, $E_{s_{n+1}}$. Finally, MLE is applied to the new reply dataset to evaluate the parameters of the network. This new BN will be stored in the agent's long term memory as s_{n+1} and will be used to predict the trustworthiness of the new informant.

1.3 Integrated Cognitive Architecture

Now that we have described both the intention reading and the trust estimation models, we are going to focus on their integration with the purpose of achieving a cognitive architecture suitable for human-robot collaborations. As described in Figure 1, the main idea is that the trust model will act as a cognitive support for the intention reading, allowing the robot to fine-tune its behavior after having decided a general course of action. In particular, the robot will initially be trained on a set of goals and will thereafter try to understand which one is being pursued by its partner. Once a confident prediction is formulated, it will offer assistance in order to achieve the shared objective. The degree of help provided is influenced by the amount of trust the robot has in that specific person: if it thinks he or she have the appropriate knowledge or skills to complete the task, then it will act as an assistive peer, on the contrary it will start behaving more like a supervisor, observing more closely the partner, correcting their mistakes and, in general, assuming more of the responsibilities to ensure that the goal is eventually reached.

The workflow we are about to discuss is shown graphically in Figure 7. The interaction starts with the robot trying to identify the partner: in case of success it fetches their trust belief BN, otherwise it generates one on-the-fly through its episodic memory. The robot is assumed to naively trust the person that has trained it, so it will possess at least one BN in its memory. After this process is completed, it will start observing the human to read his or hers intention. Once

a goal is predicted, the robot will perform a proactive trust evaluation in which it will ask itself if it expects the human to fail or succeed in the task at hand: this is done by setting X_R and Y_R as evidence and using the Message Passing algorithm to calculate the posterior probabilities for the rest of the network. At this point, the agent can use the probability distributions in nodes X_I and Y_I to infer the informant's behavior. Based on this evaluation, the robot will adopt one of two different approaches.

If the robot decides to trust the human, then it will collaborate towards the achievement of the predicted goal. Once the task is complete, it will judge the Total Output (TO) of the joint action: if the shared effort led to a successful, valid outcome its trust level towards the partner will increase, on the contrary it will decrease. If however the robot decides to distrust the partner, it will immediately inspect the Partial Output (PO), that is the portion of the task that has already been completed before an intention prediction was formulated. If the PO appears invalid, the robot will lower its trust level and will thereafter try to correct the mistake and take over the rest of the task. If instead the PO is a valid one, even if not the one which the robot had predicted, the agent will give the partner a chance to regain trust by collaborating and evaluating the TO, as described previously.

This workflow penalizes human partners who are both incapable or unwilling to contribute with an appropriate effort to the shared task, but at the same time gives distrusted people a chance to regain the trust of the robot. This is important, because failures could arise from temporary situations such as injuries or fatigue.

In an effort to include some features of Explainable AI (Hagras, 2018) into our system, the robot will try to be transparent and constantly communicate to its human partner any estimation results and any changes in its levels of trust. So, for example, if the robot doesn't trust the human to be able to accomplish a pursued goal, it will state that clearly, thus justifying its much more strict behavior. In particular, the robot will always state: the predicted goal, the estimated trust levels including any changes from trust to distrust or vice versa, its evaluation of the TO or PO and the explanation of why it believes that a task was unsuccessful. Finally, the agent will also try and justify its own errors: for example, if it realizes that the achieved goal was not the predicted one, but nevertheless was valid.

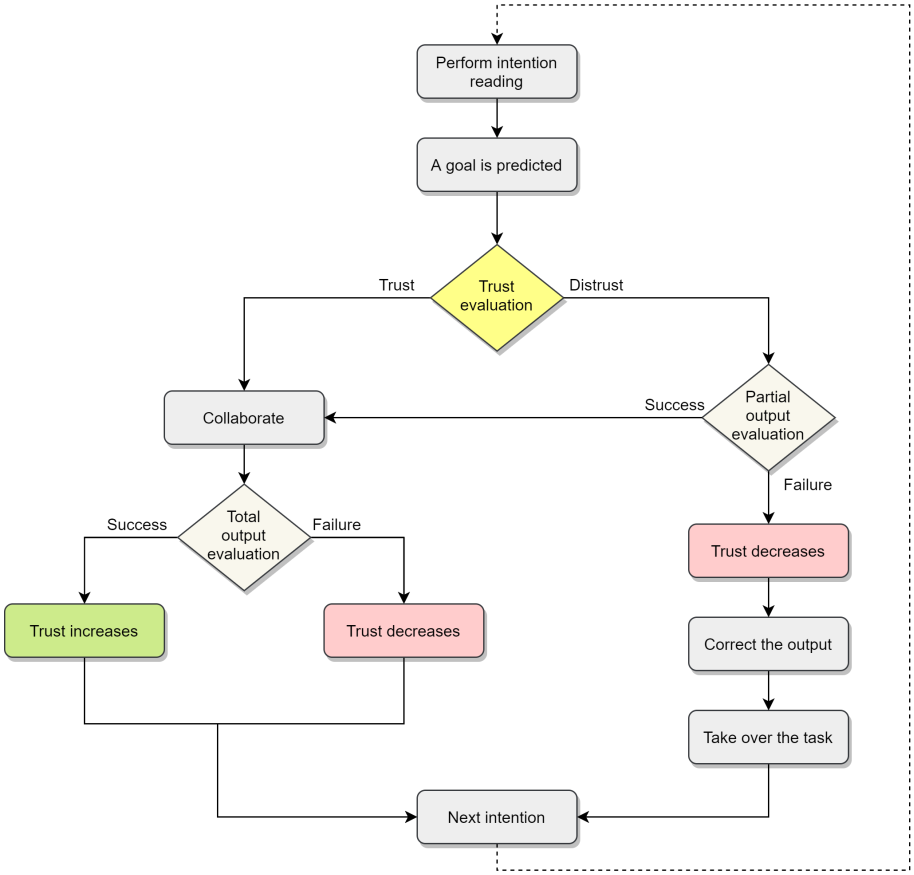

Supplemental Figure 7: The collaborative workflow. The robot reads the intention and decides if to trust or not its partner. In the former case, it provides assistance and evaluates the total output resulting from the collaboration, otherwise it adopts a more strict supervision on the human: if the partial output seems valid it gives them a chance to regain trust, otherwise it will take over the task and attempt to correct the mistakes. Related to Figure 2.

References

Aldrich, J. et al. (1997). Ra fisher and the making of maximum likelihood 1912-1922. *Statistical Science*, 12(3):162–176.

Bellman, R. (2013). *Dynamic programming*. Courier Corporation.

Cangelosi, A., Schlesinger, M., and Smith, L. B. (2015). *Developmental robotics: From babies to robots*. MIT Press.

Cao, Z., Simon, T., Wei, S.-E., and Sheikh, Y. (2016). Realtime multi-person 2d pose estimation using part affinity fields. *arXiv preprint arXiv:1611.08050*.

Cippitelli, E., Gasparrini, S., Gambi, E., and Spinsante, S. (2016). A human activity recognition system using skeleton data from RGBD sensors. *Computational intelligence and neuroscience*, 2016:21.

Douc, R. and Cappé, O. (2005). Comparison of resampling schemes for particle filtering. In *Image and Signal Processing and Analysis, 2005. ISPA 2005. Proceedings of the 4th International Symposium on*, pages 64–69. IEEE.

Erikson, E. H. (1993). *Childhood and Society*. W. W. Norton & Company.

Hagras, H. (2018). Toward human-understandable, explainable ai. *Computer*, 51(9):28–36.

Jha, S. and Busso, C. (2017). Probabilistic estimation of the driver's gaze from head orientation and position. In *2017 IEEE 20th International Conference on Intelligent Transportation Systems (ITSC)*, pages 1–6.

Lauritzen, S. L. and Spiegelhalter, D. J. (1988). Local computations with probabilities on graphical structures and their application to expert systems. *Journal of the Royal Statistical Society. Series B (Methodological)*, pages 157–224.

Malle, B. F., Moses, L. J., and Baldwin, D. A. (2001). *Intentions and intentionality: Foundations of social cognition*. MIT press.

Manzi, A., Dario, P., and Cavallo, F. (2017). A human activity recognition system based on dynamic clustering of skeleton data. *Sensors*, 17(5):1100.

Ojala, T., Pietikainen, M., and Maenpaa, T. (2002). Multiresolution gray-scale and rotation invariant texture classification with local binary patterns. *IEEE Transactions on pattern analysis and machine intelligence*, 24(7):971–987.

Patacchiola, M. and Cangelosi, A. (2017). Head pose estimation in the wild using convolutional neural networks and adaptive gradient methods. *Pattern Recognition*, 71:132 – 143.

Pelleg, D., Moore, A. W., et al. (2000). X-means: Extending k-means with efficient estimation of the number of clusters. In *ICML*, volume 1, pages 727–734.

Rekleitis, I. M. (2004). A particle filter tutorial for mobile robot localization. *Centre for Intelligent Machines, McGill University*, 3480.

Tomasello, M., Carpenter, M., Call, J., Behne, T., and Moll, H. (2005). Understanding and sharing intentions: The origins of cultural cognition. *Behavioral and Brain Sciences*, 28(5):675–691.

Vanderbilt, K. E., Liu, D., and Heyman, G. D. (2011). The development of distrust. *Child development*, 82(5):1372–1380.

Vinanzi, S., Cangelosi, A., and Goerick, C. (2020). In *2020 29th IEEE International Symposium on Robot and Human Interactive Communication (RO-MAN).*

Vinanzi, S., Goerick, C., and Cangelosi, A. (2019). Mindreading for robots: Predicting intentions via dynamical clustering of human postures. In *2019 Joint IEEE 9th International Conference on Development and Learning and Epigenetic Robotics (ICDL-EpiRob)*, pages 272–277.

Vinanzi, S., Patacchiola, M., Chella, A., and Cangelosi, A. (2019). Would a robot trust you? Developmental robotics model of trust and theory of mind. *Philosophical Transactions of the Royal Society of London B.*

Viola, P. and Jones, M. (2001). Rapid object detection using a boosted cascade of simple features. In *Computer Vision and Pattern Recognition, 2001. CVPR 2001. Proceedings of the 2001 IEEE Computer Society Conference on*, volume 1, pages I–I. IEEE.
